# Fresh fruit consumption may decrease the long‐term risk of esophageal cancer mortality: A 30‐year follow‐up study in the Linxian Dysplasia Nutrition Intervention trial (NIT)

**DOI:** 10.1111/1759-7714.13482

**Published:** 2020-05-29

**Authors:** Huan Yang, Su Zhang, Huijiao Yan, Jianbing Wang, Jinhu Fan, Youlin Qiao, Philip R. Taylor

**Affiliations:** ^1^ Department of Cancer Epidemiology, National Cancer Center/National Clinical Research Center for Cancer/Cancer Hospital Chinese Academy of Medical Sciences and Peking Union Medical College Beijing China; ^2^ Department of Epidemiology and Biostatistics The Children's Hospital, National Clinical Research Center for Child Health, Zhejiang University School of Medicine Hangzhou China; ^3^ Metabolic Epidemiology Branch, Division of Cancer Epidemiology & Genetics, National Cancer Institute National Institutes of Health Bethesda Maryland USA

**Keywords:** Esophageal squamous cell carcinoma, fresh fruit consumption, gastric cardia carcinoma, Linxian dysplasia nutrition intervention trial, noncardia carcinoma

## Abstract

**Background:**

The objective of this study was to explore the association between fresh fruit consumption and long‐term risk of upper gastrointestinal cancer (UGI) in the Linxian Dysplasia Nutrition Intervention Trial (NIT) cohort.

**Methods:**

A cohort of 3318 subjects with esophageal squamous dysplasia participated in the Linxian Dysplasia NIT in May 1985 and were followed up until 30 September 2015. Demographic characteristics, lifestyle, and history of diseases were collected at the baseline. The primary endpoint was death from esophageal squamous cell carcinoma (ESCC), gastric cardia carcinoma (GCC), and gastric noncardia carcinoma (GNCC). Hazard ratios (HRs) and 95% confidence intervals (95% CIs) were estimated using the Cox proportional hazard model.

**Results:**

In the 30‐year follow‐up, a total of 541 ESCC, 284 GCC, and 77 GNCC deaths occurred. Relative to those who never or rarely consumed fresh fruit, the risk of ESCC mortality in participants who consumed fresh fruit more than 12 times/year were significantly decreased by 37.3% (HR = 0.63, 95% CI: 0.49–0.81). In the subgroup analyses, significantly protective effects on ESCC mortality were observed especially in females (HR = 0.59, 95% CI: 0.40–0.89), non‐smokers (HR = 0.67, 95% CI: 0.48–0.94), and nondrinkers (HR = 0.69, 95% CI: 0.51–0.93).

**Conclusions:**

Consuming fresh fruit more than 12 times/year may reduce the long‐term risk of ESCC mortality in this dysplasia population, particularly in females, non‐smokers, and nondrinkers. Future studies are needed to confirm these findings.

## Introduction

Cancers of the head and neck (oral cavity, pharynx, and larynx), esophagus, and stomach are collectively called upper gastrointestinal (UGI) tract cancers. UGI cancer is one of the common types of cancer, especially in developing countries. There were approximately 1.6 million new cases of upper gastrointestinal cancer (UGI), with about 1.29 million death cases after lung cancer in the world according to GLOBOCAN 2018.^1^ Both stomach and esophageal cancers are among the top five leading causes of cancer deaths in China.^2^ Human vulnerability to UGI cancer is influenced by environmental factors including alcohol drinking and tobacco smoking for squamous cell carcinoma, and Helicobacter pylori and tobacco smoking for adenocarcinomas.^3^, ^4^ It is thought that diet plays an important role in the etiology of UGI cancer, although there is not yet any convincing evidence. Most of the evidence on the effects of diet on UGI cancer risk has been supported by observational epidemiological studies because nutritional intervention clinical trials are often unaffordable.^5^

The relationship between fruit consumption and human health was confirmed in 2002 by the World Health Organization (WHO) and the United Nations Food and Agriculture Organization (FAO).^3^ In 1997, the first World Cancer Research Fund (WCRF) panel report concluded that consumption of fruits and foods containing carotenoids and vitamin C was a protective factor of oropharyngeal cancer (OC), esophageal cancer and gastric cancer.^6^ However, the existing conclusions were based on case‐control studies or prospective investigations in a healthy population^7^, ^8^ Further studies are needed to confirm the protective effects on an undernourished population.

Linxian is a region with extremely high rates of UGI cancer in China. Previous reports in the 1980s indicated that nutritional deficiencies were common in this area, suggesting the association between nutritional deficiencies and high cancer rates.^9^ In 1985, our research team conducted two randomized, double‐blind, placebo‐controlled nutrition intervention trials (NITs) in Linxian, including dysplasia‐based (3318 participants) and general‐population based (29 584 participants) trials. The objective of NITs was to explore the effect of combinations of vitamins and minerals supplements on cancer incidence and mortality. Previous studies have consistently demonstrated a decrease in the risk of chronic disease associated with a higher intake of fruit and vegetables, especially cardiovascular disease, stroke and cancer.^5^, ^10^, ^11^, ^12^ In the Linxian general population trial, the association between fresh fruit consumption and the risk of esophageal squamous cell carcinoma (ESCC) has been extensively reported.^13^, ^14^ However, the lifestyle, nutrition and health status of the dysplasia population and general population were quite different, which may imply that the same conclusion cannot be extended to the dysplasia population. In this study, the association between fresh fruit consumption and the risk of UGI cancer mortality will be investigated in the Linxian Dysplasia Nutrition Intervention Trial (NIT) cohort.

## Methods

### Study population

**Figure 1:**
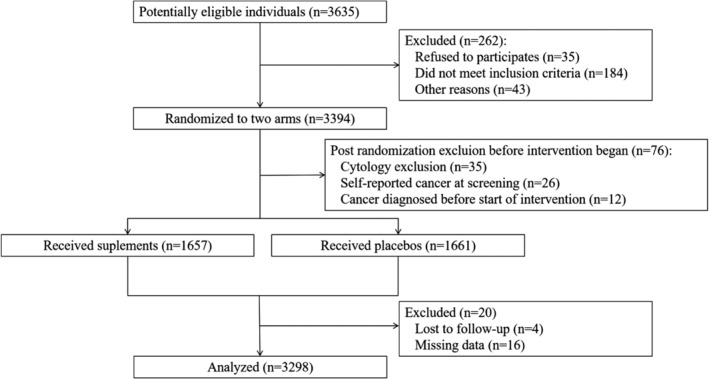
CONSORT flow diagram of the Linxian Dysplasia Population Trial Cohort.

Previous studies have reported the design and conduct of the Linxian Dysplasia NIT cohort.^15^, ^16^ In brief, the Linxian Dysplasia NIT was conducted in 1985–1991. A total of 3318 adults who were cytologically diagnosed with esophageal dysplasia were recruited from three northern communes (Yaocun, Rencun, and Donggang) in Linxian. Potential participants were eligible if they were aged between 40 and 69 years and without other debilitating disease, or regularly took any vitamins or minerals. All participants were randomly assigned to receive daily supplementation with 26 vitamins and minerals or placebos for six years until 1991. Doses of the supplement were typically 2–3 times (range, 20%–700%) the US Recommended Dietary Allowance. The enrollment of the subjects and intervention phase of this trial are further detailed in Figure 1.

### Baseline information collection

A baseline questionnaire and brief physical examination were given to collect general demographic characteristics (age, gender, region, and level of education, etc), lifestyle characteristics (smoking status, drinking status, etc), disease history and diet habits at the time of recruitment of the participants. We retrospectively investigated the consumption frequency of 10 common food categories: persimmon, moldy food, oily food, fresh vegetables, dried vegetables, sour vegetables, fresh fruit, nut, meat, and eggs. Subjects were asked how many times they had eaten the above foods in different seasons (including times/day, times/week, times/month, times/year, and never). Smokers were defined as individuals who had smoked cigarettes or used hookah or a pipe at least weekly for at least six months, and the use of alcohol was divided into no alcohol or any alcohol consumed in the previous 12 months. Dietary information included the frequency of persimmon bread, moldy bread, foods cooked in oil, meats, eggs, fruit and vegetable consumption. To avoid the bias caused by seasonal effect, we calculated the frequency of fresh fruit and vegetable consumption in winter/spring and summer/autumn seasons, respectively, and finally converted the frequency into five categories (Never, 1–3 times/year, 4–6 times/year, 7–12 times/year or more than 12 times/year).

### Follow‐up of cancer

Village Investigators with professional training visited participants to collect cancer incidence and mortality monthly data during the six‐year trial period (May 1985–April 1991). Relevant examination materials were regularly sent to the international diagnostic team, a joint panel of Chinese and American cytology pathologists and radiologists, to finally determine terminal cases for ensuring the accuracy of the study endpoint. The primary endpoints were ESCC, gastric cardia carcinoma (GCC) and gastric noncardia carcinoma (GNCC) mortality. Cancers in the most proximal 3 cm of the stomach were defined as GCC and those originating elsewhere in the stomach were defined as GNCC.^17^

### Ethics statement

The Linxian Dysplasia NIT study was approved by the Institutional Review Boards of Cancer Hospital, Chinese Academy of Medical Sciences and United States National Cancer Institute. Participants were informed of the procedure, general aim, possible benefits and risks of the study at enrollment. After confirming that they fully understood the study, they signed an informed consent form to indicate their willingness to participate. All study procedures followed the Helsinki Declaration and was regulated by the ethics committee of Cancer Hospital, Chinese Academy of Medical Sciences.

### Statistical analysis

Survival data were collected from May 1985 (the start of intervention) to each participant's death date, lost date of follow‐up, or the closure date (30 September 2015). One‐way ANOVA and chi‐square test were used to compare the differences in baseline demographic and lifestyle characteristics among the five fresh fruit consumption frequency groups. Cox proportional hazards regression models were used to calculate hazard ratios (HRs) and 95% confidence intervals (CIs) for the 30‐year effect of UGI cancer mortality, adjusting for age, gender, smoking status, alcohol drinking, BMI, fresh vegetable consumption, education level, and family cancer history at baseline. Cumulative mortality curves were estimated by the method of Kaplan‐Meier and Log‐rank tests were used to examine significance among cumulative mortality curves. Analyses were performed using SPSS 23.0. *P*‐values less than 0.05 were considered statistically significant.

## Results

After excluding 16 participants without data of diet habits data and four participants lost to follow‐up, 3298 subjects were included in the final analyses. The mean age and BMI at baseline were 53.26 ± 7.52 years and 20.35 ± 2.29 kg/m^2^, respectively. Female participants accounted for about 56.1%. Participants who reported no or rare fresh fruit consumption accounted for 30.6% of all participants, followed by participants with a consumption frequency of 4–6 times/year (25.1%) (Table 1).

**Table 1:** Baseline demographic characteristics by the frequency of fresh fruit consumption in the Linxian dysplasia nutrition intervention trial cohort

	Frequency of fresh fruit consumption (times/year)					*P*‐value*
	None	1–3	4–6	7–12	>12	
	(*n* = 1009)	(*n* = 420)	(*n* = 827)	(*n* = 398)	(*n* = 644)	
Age at interview (years, mean ± SD)	55.65 ± 6.82	52.45 ± 7.34	52.65 ± 7.33	52.59 ± 7.73	51.26 ± 7.87	**<0.01**
Body mass index (kg/m^2,^mean ± SD)	20.08 ± 2.29	20.34 ± 2.24	20.37 ± 2.32	20.56 ± 2.36	20.62 ± 2.22	**<0.01**
Consumption of fresh vegetables (times/year, mean ± SD)	591.21 ± 228.17	598.82 ± 239.66	603.96 ± 228.58	618.42 ± 229.07	648.30 ± 241.21	**<0.01**
Gender						**0.04**
Female	656 (65.0%)	267 (63.6%)	449 (54.3%)	203 (51.0%)	276 (42.9%)	
Male	353 (35.0%)	153 (36.4%)	378 (45.7%)	195 (49.0%)	368 (57.1%)	
Smoking						**<0.01**
No	773 (76.6%)	321 (76.4%)	567 (68.6%)	280 (70.4%)	399 (62.0%)	
Yes	236 (23.4%)	99 (23.6%)	260 (31.4%)	118 (29.6%)	245 (38.0%)	
Alcohol drinking						**<0.01**
No	917 (90.9%)	360 (85.7%)	674 (81.5%)	304 (76.4%)	428 (66.5%)	
Yes	92 (9.1%)	60 (14.3%)	153 (18.5%)	94 (23.6%)	216 (33.5%)	
Family cancer history						0.12
Yes	414 (41.0%)	159 (37.9%)	376 (45.5%)	201 (50.5%)	300 (46.6%)	
No	595 (59.0%)	261 (62.1%)	451 (54.5%)	197 (49.5%)	344 (53.4%)	
Education level						0.11
Non	545 (54.0%)	195 (46.4%)	360 (43.5%)	128 (32.2%)	175 (27.2%)	
<Primary education	235 (23.3%)	131 (31.2%)	248 (30.0%)	150 (37.7%)	217 (33.7%)	
≥Primary education	60 (5.9%)	44 (10.5%)	115 (13.9%)	58 (14.6%)	152 (23.6%)	
Unknown	169 (16.7%)	50 (11.9%)	104 (12.6%)	62 (15.6%)	100 (15.5%)	

* *P*‐value derived from χ2 or one‐way ANOVA tests, as appropriate, for categorical and continuous variables.

*Note: P* value was less than 0.05.

In the 30‐year follow‐up, a total of 2578 deaths occurred, including 541 with ESCC, 284 with GCC, and 77 with GNCC deaths. As compared with subjects who never or rarely consumed fresh fruit, the risk of ESCC deaths in participants who consumed fresh fruit 4–6 times/year and more than 12 times/year were significantly decreased by 22.8% (HR = 0.77, 95% CI: 0.61–0.97) and 37.3% (HR = 0.63, 95% CI: 0.49–0.81), respectively. The reduced risk of ESCC mortality was also observed after adjusting for age, BMI, gender, smoking, alcohol drinking, education level, frequency of fresh vegetable consumption, and family cancer history. However, no significant association was found between fresh fruit consumption and long‐term risk of GCC or GNCC mortality (Table 2).

**Table 2:** Crude and adjusted hazard ratios (HRs) and 95% CIs for the associations between the frequency of fresh fruit consumption and risk of UGI cancer in the Linxian dysplasia nutrition intervention trial cohort

		HRs (95% CIs)				
		None	1–3 times/year	4–6 times/year	7–12 times/year	>12 times/year
ESCC	Crude	1.000	0.859 (0.654–1.128)	**0.772 (0.614–0.970)**	0.884 (0.672–1.163)	**0.627 (0.485–0.811)**
	Age‐and gender‐adjusted	1.000	0.980 (0.745–1.290)	0.850 (0.674–1.072)	0.959 (0.727–1.265)	**0.694 (0.533–0.904)**
	Fully adjusted†	1.000	1.016 (0.772–1.338)	0.881 (0.698–1.111)	0.974 (0.736–1.289)	**0.720 (0.551–0.942)**
GCC	Crude	1.000	0.912 (0.613–1.357)	1.176 (0.868–1.592)	0.819 (0.538–1.247)	0.824 (0.580–1.172)
	Age‐and gender‐adjusted	1.000	1.070 (0.717–1.597)	1.280 (0.941–1.741)	0.861 (0.564–1.316)	0.871 (0.607–1.249)
	Fully adjusted†	1.000	1.081 (0.724–1.615)	1.313 (0.964–1.789)	0.927 (0.604–1.422)	0.913 (0.631–1.320)
GNCC	Crude	1.000	1.011 (0.529–1.929)	0.589 (0.317–1.095)	0.597 (0.271–1.316)	0.519 (0.262–1.026)
	Age‐and gender‐adjusted	1.000	1.140 (0.593–2.189)	0.639 (0.341–1.196)	0.639 (0.288–1.417)	0.558 (0.277–1.124)
	Fully adjusted†	1.000	1.200 (0.624–2.307)	0.667 (0.355–1.253)	0.633 (0.283–1.419)	0.594 (0.291–1.212)

† Adjusted for age at baseline, sex, smoking, drinking, BMI, family history of cancer, education level, frequency of fresh fruit and vegetable consumption (times/year). ESCC, esophageal squamous cell carcinoma; GCC, gastric cardia carcinoma; GNCC, gastric noncardia carcinoma

We also estimated the adjusted HRs for total UGI cancer, ESCC, GCC, and GNCC deaths after stratification by age at baseline, gender, smoking, alcohol drinking, and family cancer history (Tables 3, 4, 5). More than 12 times/year fresh fruit consumption could reduce the risk of ESCC cancer death in females, non‐smokers, and nondrinkers (HR _female_ = 0.59, 95% CI: 0.40–0.89; HR _non‐smokers_ = 0.67, 95% CI: 0.48–0.94; HR _nondrinkers_ = 0.69, 95% CI: 0.51–0.93). Significantly reduced risk of death was also observed in females and non‐smokers with GNCC (HR _female_ = 0.19, 95% CI: 0.04–0.86; HR _non‐smokers_ = 0.28, 95% CI: 0.09–0.84).

**Table 3:** Adjusted hazard ratios (HRs) and 95% CIs in subgroup analyses for the associations between the frequency of fresh fruit consumption and risk of ESCC in the Linxian dysplasia nutrition intervention trial cohort

	HRs (95% CIs)				
	None	1–3 times/year	4–6 times/year	7–12 times/year	>12 times/year
Age at interview†					
<53 years	1.000	1.031 (0.673–1.579)	0.725 (0.496–1.058)	0.941 (0.607–1.46)	0.727 (0.484–1.092)
>=53 years	1.000	0.973 (0.676–1.401)	1.021 (0.761–1.37)	1.017 (0.705–1.467)	0.729 (0.507–1.049)
Gender‡	1.000				
Female	1.000	0.89 (0.619–1.279)	0.803 (0.589–1.095)	0.76 (0.507–1.141)	**0.593 (0.396–0.889)**
Male	1.000	1.259 (0.819–1.933)	1.021 (0.713–1.462)	1.284 (0.857–1.925)	0.906 (0.619–1.326)
Smoking§	1.000				
No	1.000	1.004 (0.729–1.384)	0.895 (0.681–1.176)	0.761 (0.536–1.08)	**0.672 (0.481–0.939)**
Yes	1.000	1.101 (0.644–1.884)	0.9 (0.576–1.406)	1.586 (0.978–2.572)	0.881 (0.552–1.404)
Drinking¶	1.000				
No	1.000	1.018 (0.762–1.362)	0.834 (0.648–1.072)	0.98 (0.723–1.328)	**0.687 (0.508–0.93)**
Yes	1.000	1.131 (0.47–2.72)	1.118 (0.565–2.214)	0.916 (0.425–1.977)	0.837 (0.417–1.68)
Family cancer history††	1.000				
Yes	1.000	0.856 (0.569–1.287)	0.801 (0.579–1.107)	0.709 (0.476–1.055)	0.716 (0.497–1.032)
No	1.000	1.16 (0.797–1.69)	0.948 (0.678–1.324)	1.31 (0.883–1.944)	0.717 (0.483–1.065)

† Adjusted for BMI, gender, smoking, drinking, family history of cancer, education level, and consumption of fresh vegetables.

‡ Adjusted for age at baseline, gender, smoking, drinking, family history of cancer, education level, and consumption of fresh vegetables.

§ Adjusted for age at baseline, BMI, gender, drinking, family history of cancer, education level, and consumption of fresh vegetables.

¶ Adjusted for age at baseline, BMI, gender, smoking, family history of cancer, education level, and consumption of fresh vegetables.

†† Adjusted for age at baseline, BMI, gender, smoking, drinking, education level, and consumption of fresh vegetables.

*Note: P* value was less than 0.05.

**Table 4:** Adjusted hazard ratios (HRs) and 95% CIs in subgroup analyses for the associations between the frequency of fresh fruit consumption and risk of GCC in the Linxian dysplasia nutrition intervention trial cohort

	HRs (95% CIs)				
	None	1–3 times/year	4–6 times/year	7–12 times/year	>12 times/year
Age at interview†					
<53 years	1.000	0.808 (0.377–1.731)	1.415 (0.824–2.432)	1.026 (0.498–2.114)	1.06 (0.575–1.955)
>=53 years	1.000	1.261 (0.786–2.024)	1.249 (0.851–1.833)	0.881 (0.513–1.511)	0.837 (0.519–1.35)
Gender‡					
Female	1.000	1.073 (0.585–1.97)	1.613 (1.015–2.562)	1.111 (0.559–2.209)	1.081 (0.582–2.008)
Male	1.000	1.094 (0.641–1.868)	1.131 (0.747–1.713)	0.834 (0.481–1.444)	0.817 (0.515–1.298)
Smoking§					
No	1.000	1.085 (0.654–1.8)	1.29 (0.863–1.93)	0.794 (0.441–1.431)	0.859 (0.513–1.439)
Yes	1.000	1.076 (0.557–2.08)	1.337 (0.82–2.181)	1.178 (0.626–2.218)	0.978 (0.569–1.681)
Drinking¶					
No	1.000	1.26 (0.828–1.916)	1.408 (1.002–1.978)	1.066 (0.665–1.711)	0.887 (0.571–1.378)
Yes	1.000	0.281 (0.062–1.277)	0.916 (0.427–1.964)	0.561 (0.204–1.537)	0.79 (0.372–1.682)
Family cancer history††					
Yes	1.000	0.994 (0.52–1.901)	1.155 (0.715–1.867)	0.459 (0.21–1.004)	0.669 (0.37–1.208)
No	1.000	1.145 (0.686–1.911)	1.431 (0.954–2.147)	1.398 (0.832–2.35)	1.117 (0.697–1.79)

† Adjusted for BMI, gender, smoking, drinking, family history of cancer, education level, and consumption of fresh vegetables.

‡ Adjusted for age at baseline, gender, smoking, drinking, family history of cancer, education level, and consumption of fresh vegetables.

§ Adjusted for age at baseline, BMI, gender, drinking, family history of cancer, education level, and consumption of fresh vegetables.

¶ Adjusted for age at baseline, BMI, gender, smoking, family history of cancer, education level, and consumption of fresh vegetables.

†† Adjusted for age at baseline, BMI, gender, smoking, drinking, education level, and consumption of fresh vegetables.

**Table 5:** Adjusted hazard ratios (HRs) and 95% CIs in subgroup analyses for the associations between the frequency of fresh fruit consumption and risk of GNCC in the Linxian dysplasia nutrition intervention trial cohort

	HRs (95% CIs)				
	None	1–3 times/year	4–6 times/year	7–12 times/year	>12 times/year
Age at interview†					
<53 years	1.000	**2.631 (1.01–6.855)**	1.107 (0.415–2.951)	1.2 (0.372–3.877)	0.838 (0.28–2.506)
>=53 years	1.000	0.49 (0.145–1.658)	0.486 (0.193–1.223)	0.452 (0.131–1.562)	0.561 (0.204–1.543)
Gender‡					
Female	1.000	1.268 (0.566–2.84)	0.559 (0.236–1.322)	0.552 (0.181–1.685)	**0.19 (0.042–0.855)**
Male	1.000	0.949 (0.294–3.063)	0.774 (0.3–1.993)	0.687 (0.21–2.247)	1.085 (0.435–2.709)
Smoking§					
No	1.000	1.544 (0.774–3.078)	0.734 (0.362–1.49)	0.593 (0.234–1.503)	**0.277 (0.092–0.836)**
Yes	1.000	(−)	0.429 (0.104–1.768)	0.711 (0.139–3.646)	1.252 (0.401–3.911)
Drinking¶					
No	1.000	1.155 (0.562–2.375)	0.775 (0.402–1.494)	0.629 (0.253–1.564)	0.584 (0.257–1.325)
Yes	1.000	1.683 (0.27–10.49)	0.171 (0.017–1.704)	0.465 (0.076–2.856)	0.686 (0.131–3.605)
Family cancer history††					
>Yes	1.000	0.697 (0.227–2.146)	0.613 (0.253–1.483)	0.386 (0.109–1.368)	0.852 (0.351–2.065)
>No	1.000	1.587 (0.687–3.664)	0.691 (0.279–1.707)	1.039 (0.357–3.025)	0.32 (0.087–1.186)

† Adjusted for BMI, gender, smoking, drinking, family history of cancer, education level, and consumption of fresh vegetables.

‡ Adjusted for age at baseline, gender, smoking, drinking, family history of cancer, education level, and consumption of fresh vegetables.

§ Adjusted for age at baseline, BMI, gender, drinking, family history of cancer, education level, and consumption of fresh vegetables.

¶ Adjusted for age at baseline, BMI, gender, smoking, family history of cancer, education level, and consumption of fresh vegetables.

†† Adjusted for age at baseline, BMI, gender, smoking, drinking, education level, and consumption of fresh vegetables.

*Note: P* value was less than 0.05.

Cumulative mortality curves of ESCC, GCC and GCC and UGI cancer by frequencies of fresh fruit consumption categories are presented in Figure 1. Compared with the group of never or rarely consumed fresh fruit, a lower cumulative mortality rate of ESCC was observed in the group of consumed fresh fruit more than 12 times/year (29.9% vs. 19.5%, *P* < 0.01). The same effect was also observed in UGI cancer (41.9% vs. 30.4%, *P* < 0.01).

## Discussion

Our results showed that fresh fruit consumption may reduce the long‐term risk of ESCC mortality in the dysplasia population, particularly in females, non‐smokers, and nondrinkers. Several large prospective studies have found relatively consistently that higher fruit consumption is associated with lower all‐cause mortality and risk of chronic diseases such as chronic obstructive pulmonary disease (COPD), cardiovascular disease, diabetes, and cancer.^7^, ^8^, ^18^, ^19^, ^20^, ^21^, ^22^, ^23^, ^24^ The association between fruit consumption and digestive tract cancer mortality has been reported in previous studies.^7^, ^25^ Lifestyle is closely associated with the development of UGI cancer. Therefore, good dietary habits, specifically fresh fruit and vegetable intake, can help to prevent the onset of UGI cancer. The probable molecular mechanisms of fruit consumption against esophageal cancer may include the following: (i) Fresh fruit intake could reduce the long‐term incidence and death risk of esophageal cancer by increasing the amount of trace elements and antioxidants such as, vitamin C, carotenoids, folate, and flavonoids, which could reduce the probability of DNA damage by removing oxygen‐free radicals;^26^, ^27^ and (ii) fresh fruit consumption could increase dietary fiber intake, and this indigestible dietary nutrient composition could inhibit the malignant transformation of esophageal epithelial cells.^28^

**Figure 2:**
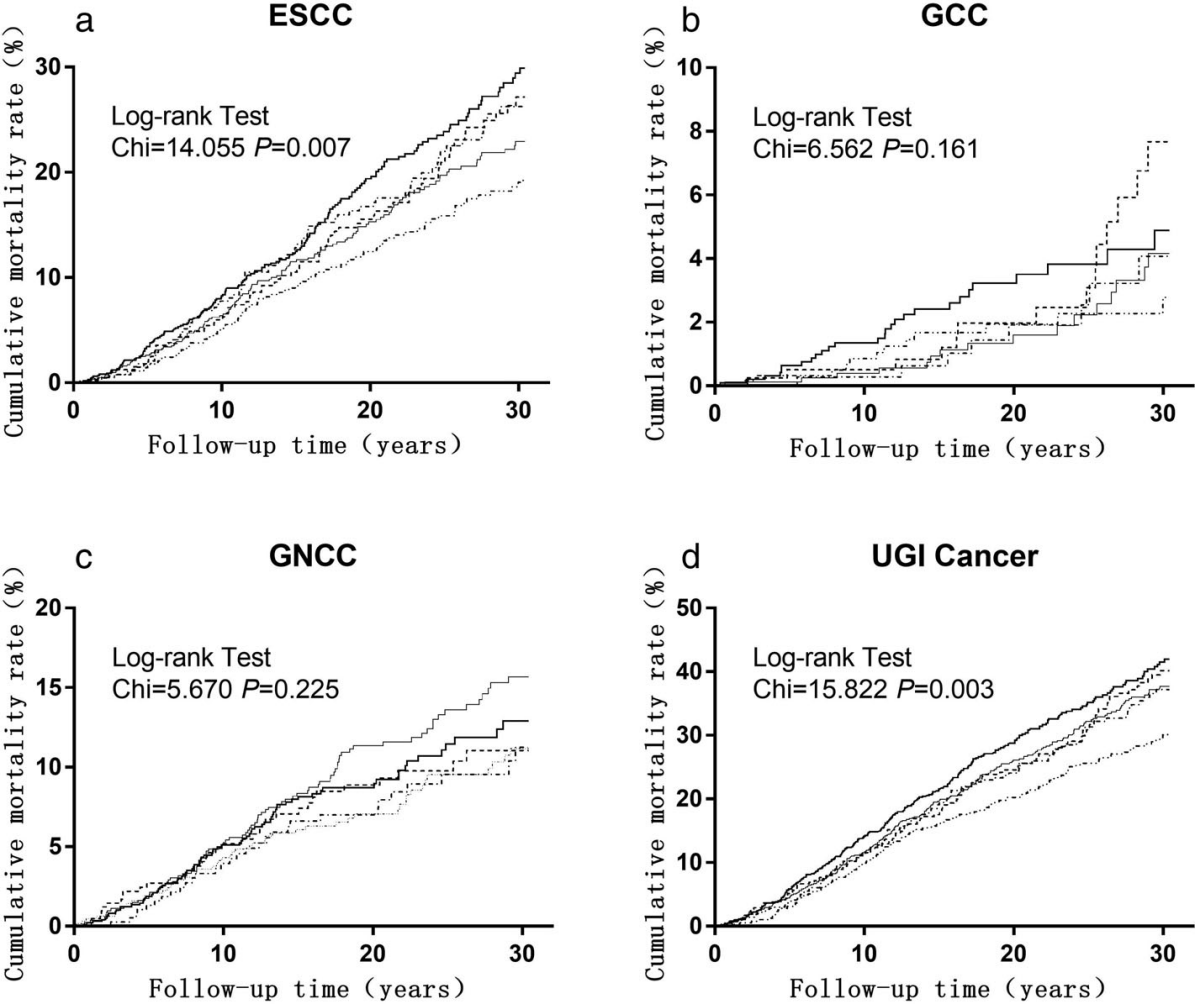
Effect of fresh fruit consumption on the cumulative incidence rate of (**a**) esophageal squamous cell carcinoma (ESCC) [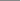 None, 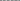 1–3 times/year, 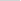 4–6 times/year, 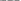 7–12 times/year, and 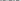 >12 times/year], (**b**) Gastric cardia carcinoma (GCC) [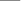 None, 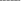 1–3 times/year, 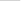 4–6 times/year, 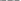 7–12 times/year, and 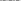 >12 times/year], Gastric noncardia carcinoma (GNCC) [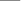 None, 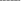 1–3 times/year, 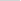 4–6 times/year, 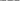 7–12 times/year, and 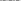 >12 times/year] and total UGI cancer (**d**) [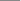 None, 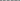 1–3 times/year, 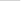 4–6 times/year, 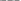 7–12 times/year, and 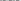 >12 times/year]. 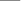 None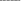:1–3 times/year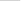: 4–6 times/year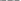: 7–12 times/year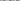: >12 times/year.

The previous diet review survey showed that fresh fruit consumption in the Linxian population had the characteristics of low intake, single variety, and great seasonal effects. The study conducted in the Linxian general population NIT cohort showed that fresh fruit consumption may decrease the long‐term risk of acquiring esophageal cancer.^13^ However, little evidence is available in the dysplasia population. Therefore, based on the Linxian dysplasia population intervention trial cohort, this study aimed to investigate whether fresh fruit intake could reduce the long‐term risk of UGI cancer death through long‐term follow‐up observations of subjects with different fresh fruit intake. Our results showed that the intake of fresh fruit may reduce the long‐term risk of ESCC mortality. According to the stratified analysis, more than 12 times/year of fresh fruit consumption could significantly reduce the long‐term risk of ESCC, particularly in women, non‐smokers, and nondrinkers, which is inconsistent with previously reported results in the general population.^14^ Wang *et al*. calculated the proportion of esophageal cancer attributable to drinking, smoking, low vegetable and fruit consumption. The esophageal cancer incidence and mortality data was based on the third national death cause survey and population‐based cancer registries in China. The results showed that these four risk factors could increase esophageal cancer incidence and mortality risk.^29^ However, the present findings suggested that the effects of smoking and drinking, once other lifestyles such as dietary habit were taken into account, was of much less significance. A case‐control study evaluated the joint effect of fruit consumption combined with drinking and smoking on esophageal cancer. The study showed that there was a multiplicative interaction between fruit consumption and smoking and alcohol drinking. After adjusting for age, gender, socioeconomic status, education level, and other confounders, fruit consumption could still decrease esophageal cancer risk associated with smoking and alcohol drinking.^30^ The attenuation of the relative risk for smoking and alcohol drinking by adjusting for fresh fruit intake suggested that part of the risk attributed to smoking and drinking was due to the deficiency of protective foods. In the NIT general population cohort, only 16.4% of male smokers never or rarely ate fresh fruit, while others usually consumed fresh fruit more than once per month. During the 30‐year follow‐up, 891 ESCC deaths occurred in male smokers, which accounted for 10.1% of the total number of NIT general population.^14^ In the dysplasia cohort, more than 80% of participants ate fruit less than 12 times/year. However, 17.3% of subjects died of esophageal cancer. Therefore, the higher fresh fruit intake, as well as the lower mortality rate of ESCC in male smokers in NIT general population cohort, could lead to the observed significant protective effect of fruits on ESCC death. In the dysplasia population, the frequency of fruit consumption was too low to offset the harm caused by smoking and alcohol drinking, which may explain why no significant protective effect was seen among smokers and drinkers (Fig 2).

Men usually have a higher risk of developing ESCC in China,^31^ and this may be related to the lifestyle between different genders. A previous study indicated that in China, smoking and alcohol drinking were associated with an esophageal cancer risk among men, but not among women in the high‐risk area.^32^ In the dysplasia cohort, smokers and drinkers accounted for only 0.6% and 7.8% in female subjects, respectively, while 64.8% and 33.3% of male subjects had a history of smoking and alcohol drinking, respectively. This result means that women could have a better lifestyle and be more likely to have a lower risk of ESCC death, which may lead to increased survival and bias and overestimated protection caused by fresh fruit consumption. Protective effects were also seen in older subjects, males, smokers, and drinkers, although the difference was not statistically significant. The probable reason may be the larger proportion of cases among older subjects, males and smokers. Unmeasured confounders could also lead to the underestimated association between smoking and UGI cancer. A further study will be conducted to explore the possible reasons.

This study had several advantages such as large number of cancer cases, long‐term follow‐up, and low rate of lost to follow‐up. At the same time, early research showed that the fresh fruit intake of the population in Linxian did not change significantly during the 15 years,^13^ providing a basis for the analysis of this study. However, our study still had some limitations. First, it was difficult to make the pathological diagnosis in Linxian during the baseline survey period, so we only included individuals with a cytological diagnosis of esophageal squamous dysplasia, which indicated that the generalization of our results to other populations remains uncertain. Second, fruit consumption frequency was assessed through a baseline diet questionnaire. Accurate quantitative measurements were not possible. As the follow‐up time extended, fresh fruit intake and other lifestyle habits such as smoking and alcohol drinking also changed, which may lead to erroneous estimates of the association between fresh fruit intake and long‐term risk of UGI cancer deaths and the existence of recall bias. Third, apart from vegetable intake, other dietary factors were not considered in this study, so we were unable to evaluate the interaction between other dietary factors and fresh fruit intake. Socioeconomic status (SES), which could be predicted with education level,^33^ was an unmeasured factor. Although we included education level into our analyses, we still could not confirm whether this indicator can completely replace socioeconomic status.

In summary, consuming fresh fruit more than 12 times/year may reduce the long‐term risk of ESCC mortality in this dysplasia population, especially in females, non‐smokers, and nondrinkers. Future studies are needed to confirm these findings. If this association is largely causal and effective measures can be implemented to increase fresh fruit consumption, the risk of ESCC mortality can be significantly reduced.

## Disclosure

No authors report any conflict of interest.

## References

[1] Bray F , Ferlay J , Soerjomataram I , Siegel RL , Torre LA , Jemal A . Global cancer statistics 2018: GLOBOCAN estimates of incidence and mortality worldwide for 36 cancers in 185 countries. CA Cancer J Clin 2018; 68 (6): 394–424. 30207593 10.3322/caac.21492

[2] Chen W , Zheng R , Baade PD *et al* Cancer statistics in China, 2015. CA Cancer J Clin 2016; 66 (2): 115–32. 26808342 10.3322/caac.21338

[3] Johnson IT . Understanding the association between diet and nutrition in upper gastrointestinal cancer. Expert Rev Gastroenterol Hepatol 2015; 9 (11): 1347–9. 26366708 10.1586/17474124.2015.1088383

[4] Enzinger PC , Mayer RJ . Esophageal cancer. N Engl J Med 2003; 349 (23): 2241–52. 14657432 10.1056/NEJMra035010

[5] Abnet CC , Corley DA , Freedman ND , Kamangar F . Diet and upper gastrointestinal malignancies. Gastroenterology 2015; 148 (6): 1234–43. 25680671 10.1053/j.gastro.2015.02.007 PMC4414068

[6] WCRF/AICR . Food, Nutrition, Physical Activity and the Prevention of Cancer: A Global Perspective. American Institute for Cancer Research, Washington DC 2007.

[7] Boffetta P , Couto E , Wichmann J *et al* Fruit and vegetable intake and overall cancer risk in the European Prospective Investigation into Cancer and Nutrition (EPIC). J Natl Cancer Inst 2016; 18 (4): 361–73. 10.1093/jnci/djq072 20371762

[8] Jochems SHJ , Osch FHMV , Reulen RC , Hensbergen MV , Zeegers MP . Fruit and vegetable intake and the risk of recurrence in patients with non‐muscle invasive bladder cancer: A prospective cohort study. Cancer Causes Control 2018; 29 (17): 1–7. 29667104 10.1007/s10552-018-1029-9 PMC5938309

[9] Yang CS , Sun Y , Yang QU *et al* Vitamin A and other deficiencies in Linxian, a high esophageal cancer incidence area in northern China. J Natl Cancer Inst 1984; 73 (6): 1449–53. 6595453

[10] Silvera SAN , Mayne ST , Gammon MD *et al* Diet and lifestyle factors and risk of subtypes of esophageal and gastric cancers: Classification tree analysis. Ann Epidemiol 2014; 24 (1): 50–7. 24239095 10.1016/j.annepidem.2013.10.009 PMC4006990

[11] Wang JB , Fan JH , Dawsey SM *et al* Dietary components and risk of total, cancer and cardiovascular disease mortality in the Linxian nutrition intervention trials cohort in China. Sci Rep 2016; 6: 22619. 26939909 10.1038/srep22619 PMC4778051

[12] Du H , Li L , Bennett D *et al* Fresh fruit consumption and major cardiovascular disease in China ‐ NEJM. N Engl J Med 2016; 374 (14): 1332–43. 27050205 10.1056/NEJMoa1501451 PMC4896382

[13] Yang Z , Wang S , Liang H , Yu P , Fan J , Qiao Y . Fresh fruit consumption may decrease the long‐term risk of acquiring esophageal cancer. Chin J Clin Oncol 2016; 43: 808–13.

[14] Liang H , Wang S‐M , Yang Z *et al* Fresh fruit intake and long‐term risk of esophageal cancer among male smokers: Finding from the Linxian nutrition intervention trials after 30 years of follow up. China Cancer 2017; 26 (12): 953–9.

[15] Mark SD , Wang W , Fraumeni JF *et al* Lowered risks of hypertension and cerebrovascular disease after vitamin/mineral supplementation: The Linxian nutrition intervention trial. Am J Epidemiol 1996; 143 (7): 658–64. 8651227 10.1093/oxfordjournals.aje.a008798

[16] Zhang S , Yu P , Wang JB , Fan JH , Qiao YL , Taylor PR . Association between tooth loss and upper gastrointestinal cancer: A 30‐year follow‐up of the Linxian dysplasia nutrition intervention trial cohort. Thorac Cancer 2019; 10 (4): 966–74. 30883021 10.1111/1759-7714.13037 PMC6449253

[17] Murphy G , Fan JH , Mark SD *et al* Prospective study of serum cysteine levels and oesophageal and gastric cancers in China. Gut 2011; 60 (5): 618–23. 21242262 10.1136/gut.2010.225854 PMC3428021

[18] Oyebode O , Gordon‐Dseagu V , Walker A , Mindell JS . Fruit and vegetable consumption and all‐cause, cancer and CVD mortality: Analysis of health survey for England data. J Epidemiol Community Health 2014; 68 (9): 856–62. 24687909 10.1136/jech-2013-203500 PMC4145465

[19] Schwingshackl L , Schwedhelm C , Hoffmann G *et al* Food groups and risk of colorectal cancer. Int J Cancer 2018; 142 (9): 1748–58. 29210053 10.1002/ijc.31198

[20] Steevens J , Schouten LJ , Goldbohm RA , van den Brandt PA . Vegetables and fruits consumption and risk of esophageal and gastric cancer subtypes in The Netherlands cohort study. Int J Cancer 2011; 129 (11): 2681–93. 21960262 10.1002/ijc.25928

[21] Du H , Li L , Bennett D *et al* Fresh fruit consumption and all‐cause and cause‐specific mortality: Findings from the China Kadoorie biobank. Int J Epidemiol 2017; 46 (5): 1444–55. 28449053 10.1093/ije/dyx042 PMC5837264

[22] Szmidt MK , Kaluza J , Harris HR , Linden A . Long‐term dietary fiber intake and risk of chronic obstructive pulmonary disease: A prospective cohort study of women. Eur J Nutr 2019. https://doi.org/10.1007/s00394‐019‐02038‐w. 10.1007/s00394-019-02038-w PMC7351821 31280344

[23] Du H , Li L , Bennett D *et al* Fresh fruit consumption and major cardiovascular disease in China. N Engl J Med 2016; 374 (14): 1332–43. 27050205 10.1056/NEJMoa1501451 PMC4896382

[24] Du H , Li L , Bennett D *et al* Fresh fruit consumption in relation to incident diabetes and diabetic vascular complications: Findings from the China Kadoorie biobank study. PLOS Med 2017; 14 (4): e1002279. 28399126 10.1371/journal.pmed.1002279 PMC5388466

[25] Wang X , Ouyang Y , Liu J *et al* Fruit and vegetable consumption and mortality from all causes, cardiovascular disease, and cancer: Systematic review and dose‐response meta‐analysis of prospective cohort studies. BMJ 2014; 349: g4490. 25073782 10.1136/bmj.g4490 PMC4115152

[26] Chung MY , Lim TG , Lee KW . Molecular mechanisms of chemopreventive phytochemicals against gastroenterological cancer development. World J Gastroenterol 2013; 19 (7): 984–93. 23467658 10.3748/wjg.v19.i7.984 PMC3582010

[27] Wang A , Zhu C , Fu L *et al* Citrus fruit intake substantially reduces the risk of esophageal cancer: A meta‐analysis of epidemiologic studies. Medicine 2015; 94 (39): e1390. 26426606 10.1097/MD.0000000000001390 PMC4616874

[28] Vingeliene S , Chan DSM , Aune D *et al* An update of the WCRF/AICR systematic literature review on esophageal and gastric cancers and citrus fruits intake. Cancer Causes Control 2016; 27 (7): 837–51. 27153845 10.1007/s10552-016-0755-0 PMC4923099

[29] Wang JB , Fan JH , Liang H *et al* Attributable causes of esophageal cancer incidence and mortality in China. PLOS One 2012; 7 (8): e42281. 22876312 10.1371/journal.pone.0042281 PMC3410925

[30] Liu S , Huang L‐P , Lin Z , Yang H‐M , Zhang Z‐H , Lu W‐T . Joint effect of fruit consumption with smoking and alcohol drinking on esophageal cancer: A case‐control study. Chin J Public Health 2019; 35 (6): 731–4.

[31] Domper Arnal MJ , Ferrández Arenas Á , Lanas AÁ . Esophageal cancer: Risk factors, screening and endoscopic treatment in Western and eastern countries. World J Gastroenterol 2015; 21 (26): 7933–43. 26185366 10.3748/wjg.v21.i26.7933 PMC4499337

[32] Wu M , Zhao J‐K , Zhang Z‐F *et al* Smoking and alcohol drinking increased the risk of esophageal cancer among Chinese men but not women in a high‐risk population. Cancer Causes Control 2011; 22 (4): 649–57. 21321789 10.1007/s10552-011-9737-4 PMC3059761

[33] Cundiff J , Uchino B , Smith T , Birmingham W . Socioeconomic status and health: Education and income are independent and joint predictors of ambulatory blood pressure. J Behav Med 2015; 38 (1): 1–8. 23645146 10.1007/s10865-013-9515-8

